# Proinflammatory gene and protein expression alterations in human limbal aniridia fibroblasts

**DOI:** 10.1371/journal.pone.0337114

**Published:** 2025-12-04

**Authors:** Julia Sarah Alexandra Zimmermann, Simon Trusen, Fabian Norbert Fries, Zhen Li, Ning Chai, Berthold Seitz, Shweta Suiwal, Maryam Amini, Nóra Szentmáry, Tanja Stachon

**Affiliations:** 1 Dr. Rolf M. Schwiete Center for Limbal Stem Cell and Aniridia Research, Saarland, University, Homburg/Saar, Germany; 2 Department of Ophthalmology, Saarland University Medical Center, Homburg/Saar, Germany; Cedars-Sinai Medical Center, UNITED STATES OF AMERICA

## Abstract

Aniridia-associated keratopathy (AAK) results from the paired box 6 (PAX6) gene haploinsufficiency, leading to depletion of limbal epithelial stem cells, persistent inflammation and a gradual loss of vision. Although fibroblasts are acknowledged as sentinel cells initiating corneal inflammation, their contribution in the chronic inflammatory state of AAK remains unexplored. Our study aims to compare PAX6 and inflammatory cytokine expression in limbal fibroblast cells (LFCs) of corneal donors and aniridia patients (AN-LFCs). Furthermore, we subjected LFCs and AN-LFCs to LPS and cobalt chloride (CoCl_2_), to investigate specific inflammatory reactions. Following isolation and culture, primary LFCs (*n* = 7) and AN-LFCs (*n* = 7) were subjected to a 24-hour treatment with *E. coli* LPS or a 48-hour exposure to CoCl_2_ as a chemical oxidative stress (OS) inducer. Subsequently, PAX6 as well as IL-1β, IL-6, TNF-α, and VEGF gene expression was examined by qPCR. Corresponding protein levels were assessed by ELISA from the cell culture supernatants. AN-LFCs exhibited similar PAX6 mRNA levels as normal LFCs (*p* ≥ 0.38). Notably, LPS treatment (*p* ≤ 0.02), but not CoCl_2_, significantly enhanced PAX6 expression. Untreated AN-LFCs showed higher IL-6 mRNA (*p* = 0.002) and protein levels (*p* = 0.009) but lower TNF-α protein expression (*p* = 0.016) than LFCs. When exposed to LPS, AN-LFCs exhibited higher IL-1β mRNA (*p* = 0.0008), as well as IL-6 protein expression (*p* = 0.029) than normal LFCs. Under OS, an elevated IL-6 protein (*p* = 0.010) was observed in AN-LFCs compared to LFCs. Our study revealed inflammatory gene and protein expression alterations in AN-LFCs, that could be involved in the stromal niche perturbations seen in AAK.

## Introduction

Congenital aniridia, a rare genetic disorder, is often associated with severe malformations in almost all sections of the eye [1]. In about 90% of cases, it can be attributed to haploinsufficiency of the paired box 6 (PAX6) gene [2]. It exhibits partial progression and almost always leads to a reduction in visual acuity, which can range from slight impairments to complete blindness [3,4].

Despite being named after the partial to subtotal absence of the iris [5,6], this symptom makes only a minimal contribution to the patient’s complaints. However, the real threats to vision lie in congenital macular hypoplasia, juvenile cataract formation, secondary glaucoma with the risk of optic atrophy, and progressive corneal changes, that, being observed in nearly every patient, can culminate in the development of aniridia associated keratopathy (AAK) [1].

Key features of AAK involve a limited epithelial regenerative capacity, leading to persistent epithelial defects and eventual scarring of the ocular surface, as well as an increasing conjunctivalization of the cornea [6]. In the epithelium-adjacent stroma, fibrotic remodeling occurs, giving rise to subepithelial pannus formation. These changes coincide with corneal opacity and subsequent visual loss [4,7].

In the research on AAK the initial scientific focus was directed to the limbal epithelial stem cells (LESCs) residing in the palisades of Vogt [8–10]. Their role is to replace shed epithelial cells via targeted proliferation, followed by migration to the center of the cornea along with progressive differentiation to form a mature epithelium with solid cell associations [4,11].

Besides the manifestations of limbal stem cell deficiency, characterized by a destructive loss of LESCs due to thermal or chemical burns, surgical procedures, Steven-Johnson syndrome, and other circumstances, a second category is distinguished. In this category, a similar phenotypic presentation develops gradually without direct damage to the LESCs. Congenital aniridia is among the conditions in this category. Some authors suggest that dysfunctions in the limbal stroma, stemming from various causes, play a significant role in driving this type of limbal stem cell deficiency [12,13], where a chronic state of inflammation is a common element [13].

In AAK, the lack of epithelial differentiation markers results in the development of intercellular clefts and increased epithelial fragility [3,14,15]. However, there is conflicting evidence regarding the impact of PAX6 deficiency on the proliferative and migratory behavior of the epithelial cells, which is pertinent in the context of wound healing. Interestingly, PAX6 haploinsufficient epithelial cells exhibit a reduced rate of cell division in monoculture experiments [16,17], while there is typically an expedited proliferation and wound healing response in whole organ specimens [14,18]. Leiper et al. propose that the influence of inflammatory processes in the corneal stroma accelerates these events, potentially explaining the observed contradictions [17]. Notably, immune cell infiltration is observed at a very early point in time when the limbal structure is still fully intact [7,19]. Further speculation suggests that the enhanced proliferation of LESC over several years may lead to the observed depletion of stem cells [6,20].

Stromal fibroblasts act as sentinel cells of the central cornea by releasing cytokines upon stimulation, initiating an inflammatory response in the otherwise immunologically quiescent and immune-privileged cornea [21]. Given their minimal PAX6 expression, their role in AAK pathogenesis has been poorly researched so far [19].

In the present study, our aim is to investigate whether limbal fibroblasts from aniridia patients and unaffected corneal donors differ in the expression and secretion of cytokines that could contribute to the chronic inflammatory state in aniridia. Additionally, we will assess how limbal fibroblasts from both groups respond to specific inflammatory stimuli. While lipopolysaccharides (LPS), as components of bacterial cell membranes, are commonly used in experimental studies to induce inflammation [22], *in vivo*, other processes might be responsible for the characteristic inflammatory state in AAK. For instance, Ou and colleagues demonstrated a significantly elevated oxidative stress (OS) level alongside chronic wound conditions in PAX6 haploinsufficient mice [23]. OS has already been identified as a pathognomonic factor in a variety of ocular diseases, causing not only direct tissue damage, but also the secretion of inflammatory cytokines by immune-competent cells [24]. OS induction can be achieved, among other methods, chemically through cobalt chloride (CoCl_2_) [25]. In addition to a quiescent environment, where no inflammatory stimuli were present, we are examining the response of primary limbal fibroblasts to exposure to LPS and CoCl_2_ on the inflammatory cytokines interleukin (IL)-1β, IL-6, tumor necrosis factor-α (TNF-α), and vascular endothelial growth factor (VEGF).

## Materials and methods

### Ethics approval and consent to participate

This study was approved by the Ethics committee of Saarland/Germany (No. 178/22). The declaration of Helsinki was respected and written informed consent was obtained from all adult donors, and in the case of minors, consent was obtained from parents in accordance with applicable ethical guidelines and regulatory requirements for the use of their biological specimens in research. The archived samples were accessed for research purposes between 03.11.2022 and 31.07.2023. The authors accessed anonymized specimens that contained no personally identifiable donor information at any point throughout the study.

### Cell culture

Limbal tissues were intraoperatively obtained as biopsies from 7 patients with congenital aniridia in the Department of Ophthalmology at Saarland University Medical Center. For comparison, post-mortem biopsies from the limbal region of 7 corneal donors, attributed for research purposes, were provided by the LIONS Cornea Bank Saar-Lor-Lux, Trier/Westpfalz. Further information on the patients and donors is listed in Table 1 and 2. All tissue samples measuring 1–2 mm^2^ were further processed for cell cultivation within 24 h after collection.

**Table 1:** Descriptive data of corneal donors.

Donor	Age (years)	Gender
LFC 1	83	Female
LFC 2	76	Male
LFC 3	78	Male
LFC 4	85	Male
LFC 5	98	Female
LFC 6	65	Male
LFC 7	77	Male

LFC: Limbal fibroblast cells.

**Table 2:** Patient characteristics.

Patient	Age (years)	Gender	AAK grade^†^
AN-LFC 1	37	Male	4
AN-LFC 2	16	Female	3
AN-LFC 3	65	Male	4
AN-LFC 4	78	Male	4
AN-LFC 5	30	Female	5
AN-LFC 6	48	Female	4
AN-LFC 7	59	Female	4

^†^Aniridia associated keratopathy (AAK) stages according to Lagali et al. [26].

AN-LFCs: Aniridia limbal fibroblast cells.

Cell culture work was conducted under sterile conditions. Biopsies were initially rinsed with Dulbecco’s Phosphate Buffered Saline (DPBS) (Sigma-Aldrich, St. Louis, USA) and then incubated for 24 h at 37°C in Gibco^®^ Keratinocyte SFM (Thermo Fisher Scientific, Waltham, USA) containing 2.0 mg/ml collagenase A (Roche Diagnostics GmbH, Mannheim, Germany) to isolate the cells from the tissue. This enzymatic digestion method is primarily recommended for processing limbal samples, as it effectively cleaves the supportive structure of the limbal stroma, leaving limbal epithelial cells in interconnected cell clusters through the preserved basement membrane [27,28]. Thereafter these cell clusters were effectively separated from isolated keratocytes using a Flowmi^™^ Cell Strainer with a pore size of 40 µm (Bel-Art SP Scienceware, Wayne, USA). The flow-through containing the limbal keratocytes was centrifuged at 800 g for 5 min. The supernatant was decanted, and the cell pellet was resuspended in 1 ml DPBS and seeded in a T75 cell culture flask with 13 ml of basic culture medium consisting of Dulbecco’s modified Eagle’s medium (DMEM/F12) (Thermo Fisher Scientific, Waltham, USA), supplemented with 5% fetal calf serum (FCS) (Thermo Fisher Scientific, Waltham, USA) and 1% penicillin-streptomycin (P/S) (Sigma-Aldrich, St. Louis, USA). Cultivation was carried out in an incubator at 37°C with 95% relative humidity and 5% CO_2_ atmosphere. The medium was exchanged every 3 days until achieving confluence. Due to the presence of FCS, keratocytes underwent differentiation into fibroblasts, and will thus be subsequently referred to as “limbal fibroblast cells” (LFCs) or “aniridia limbal fibroblast cells” (AN-LFCs).

Confluent cell cultures were harvested using Trypsin-EDTA (0.05% trypsin/0.02% EDTA, Sigma-Aldrich, St. Louis, USA) and transferred into new T75 culture flasks for further passages. Experiments were conducted employing primary fibroblasts from passages 6–10.

### Lipopolysaccharide exposure

To simulate an infectious inflammatory state, fibroblasts were exposed to *Escherichia coli* lipopolysaccharides (LPS). Following the final cell passage, the culture medium was gently aspirated at 90% confluence, and the cell monolayer was carefully rinsed with DPBS. Subsequently, all LFCs and AN-LFCs were cultured for 24 h in the presence of 0 µg/ml and 17.5 µg/ml LPS (O26:B6, Sigma-Aldrich, St. Louis, USA) in 13 ml serum-free medium composed of DMEM/F12, 1% P/S, and 1% Insulin-Transferrin-Selenium (ITS) supplement (Sigma-Aldrich, St. Louis, USA) at 37°C, in the incubator. The 24-hour exposure period was chosen as it has been proven effective in triggering LPS-induced inflammation [29,30]. The withdraw of FCS aimed to prevent potential interference of serum components with protein measurements from cell culture supernatants.

### CoCl_2_ exposure

To induce hypoxic conditions, all LFCs and AN-LFCs were treated with 0 µM and 75 µM CoCl_2_ (Sigma-Aldrich, St. Louis, USA) for 48 h. CoCl_2_ serves as an inhibitor of prolyl hydroxylases that chemically simulates hypoxia, leads to oxidative stress and evokes an inflammatory response. The 48-hour exposure duration was chosen based on previous experiences of our research group with the induction of OS using CoCl_2_ [31,32].The procedure paralleled the steps of LPS exposure.

### Collection of supernatant and cell harvesting

For protein determination from the cell culture supernatant, the culture medium was carefully aspirated after LPS or CoCl_2_ exposure, followed by centrifugation at 1500 RPM for 5 min to remove cellular detritus. From the supernatant, 4 ml per flask was collected and stored at –80°C.

The cell monolayer was then rinsed with DPBS, harvested with Trypsin-EDTA, and pelletized. The dried cell pellets were also frozen at –80°C for further use.

### RNA isolation and cDNA synthesis

RNA isolation from cell pellets was performed using the Total RNA Purification Plus Micro Kit (Norgen Biotek Corp., Thorold, Canada) according to the manufacturer’s instructions. RNA quantity was determined via UV/VIS spectrophotometry (Analytik Jena AG, Jena, Germany). The eluted RNA was then preserved at –80°C until cDNA synthesis.

cDNA synthesis was conducted utilizing the OneTaq^®^ RT-PCR Kit (New England Biolabs Inc., Ipswich, USA) following the provided protocol. 500 ng RNA was applied as a template for cDNA amplification. The synthesized cDNA was stored at –20°C for further use.

### Quantitative PCR

For quantitative PCR (qPCR), validated primers for a SYBR Green-based method from Qiagen GmbH (Hilden, Germany) were used, which are listed in Table 3. The reaction mix was prepared with 1 µl specific primer solution, 5 µl AceQ^®^ qPCR SYBR Green Master Mix (Vazyme Biotech, Nanjing, China) and 3 µl of nuclease-free water, which was supplemented with 1.2 µl cDNA per well. The qPCR was carried out in 40 cycles under the following amplification conditions using the QuantStudio 5 Real-Time PCR System (Thermo Fisher Scientific, Waltham, USA): Initial denaturation at 95°C for 10 s, primer annealing at 60°C for 30 s, elongation at 95°C for 15 s. Cooling and heating were performed at a rate of 1.6°C/s.

**Table 3:** Primer pairs for qPCR.

Targeted cDNA	Referred as	Qiagen Cat. No.	Amplicon size (bp)
Hs_PAX6_1_SG	PAX6	QT00071169	113
Hs_IL1b_1_SG	IL-1β	QT00021385	117
Hs_IL6_1_SG	IL-6	QT00083720	107
Hs_TNF_1_SG	TNF-α	QT00029162	98
Hs_VEGFA_1_SG	VEGF	QT01010184	150, 204, 222, 273
Hs_GUSB_1_SG	GUSB	QT00046046	96
Hs_TBP_1_SG	TBP	QT00000721	132

bp: base pairs.

The measured cycle threshold (Ct) values were normalized to the average expression level of the reference genes TATA binding protein (TBP) and β-glucuronidase (GUSB) (ΔCt) and compared to the average ΔCt value of untreated LFCs (0 µg/ml LPS or 0 µM CoCl_2_) using the ΔΔCt method [33]. ΔCt was used for statistical analysis. The graphical representation was based on the fold change (FC = 2^-ΔΔCt^).

### Protein quantification

To normalize the quantified cytokines in the supernatant, the total protein concentrations of the corresponding cell pellets were employed. Frozen cell pellets were lysed with RIPA buffer (Sigma-Aldrich, St. Louis, USA), and protein concentrations were assessed through the Pierce^™^ BCA Protein Assay Kit (Thermo Fisher Scientific, Waltham, USA). The measurement was conducted using the Tecan Infinite F50 Absorbance Microplate Reader (Tecan Group AG, Männedorf, Switzerland) at 560 nm wavelength. Bovine Serum Albumin served as a reference standard.

### ELISA

Cytokine concentrations were determined from cell culture supernatants using enzyme-linked immunosorbent assay (ELISA). Employing a sandwich ELISA principle, IL-1β, IL-6, TNF-α, and VEGF levels were quantified in LFC and AN-LFC cultures after 24 h of LPS treatment or 48 h of CoCl_2_ exposure using DuoSet^®^ ELISA Kits from R&D Systems Europe, Ltd. (Abingdon, UK). For each well, a 100 µl sample of the collected cell culture supernatant was utilized for assessment. The quantitative detection of cytokines was performed by Tecan Infinite F50 Absorbance Microplate Reader at 450 nm wavelength, based on a standard curve. The measured cytokine concentration in pg/ml was normalized to the previously determined total protein concentration in mg/ml. The quotient (pg/mg) was subjected to statistical analysis.

### Statistical analysis

Statistical analysis and graphical representation of results were performed using GraphPad Prism software (Version 9.5.1). Differences between LFCs and AN-LFCs, which originated from different donors, were assessed using unpaired two-tailed t-tests (Welch’s t-test). For within-group comparisons, paired two-tailed t-tests for repeated measures were used, as cells derived from the same donor were exposed to different treatment conditions within each biological replicate. *p* < 0.05 was considered statistically significant. Results were presented as arithmetic means with corresponding standard deviations (S1 Table). Differing from this, the qPCR fold changes (2^-ΔΔCt^), representing a ratio between the calculated means of the control group and the experimental condition, were presented as geometric means with corresponding geometric standard deviations (S2 Table), given that a geometric mean is a more suitable representation for ratios.

## Results

### PAX6 expression in LFCs and AN-LFCs under inflammatory conditions and oxidative stress

Despite untreated AN-LFCs in both sub-experiments displaying only 57% (FC_0 µg/ml LPS_ = 0.574) and 71% (FC_0 µM CoCl2_ = 0.712) of the mRNA levels of normal LFCs, the difference between LFCs and AN-LFCs reached no statistical significance at any of the studied conditions.

**Fig 1:**
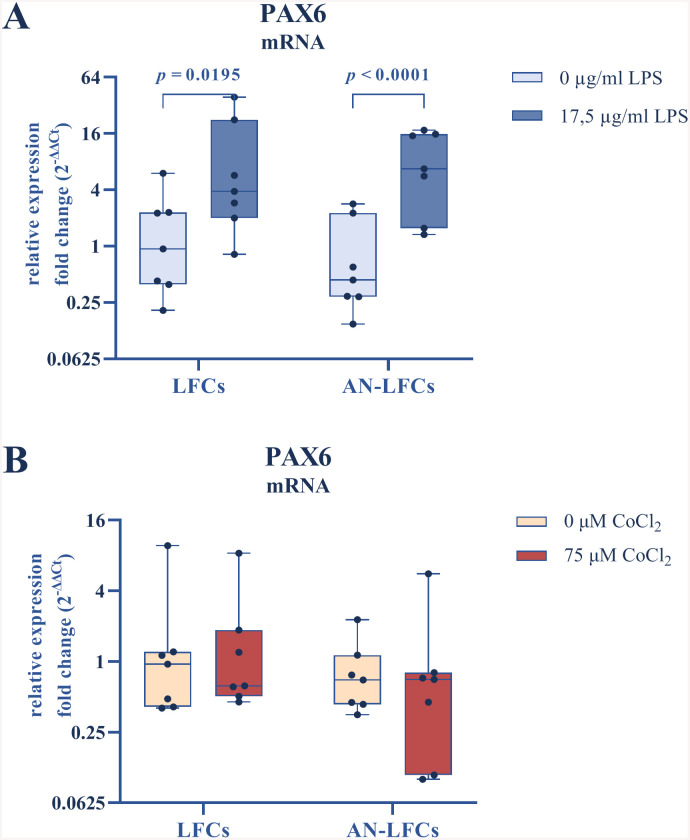
Effect of LPS-induced inflammation (A, blue) and CoCl_2_-triggered oxidative stress (B, red) on PAX6 mRNA expression. The induction of an inflammatory response was achieved by applying 17.5 µg/ml E. coli LPS over a 24 h period. Oxidative stress was simulated by administering 75 µM CoCl_2_ over 48 **h.** All results are presented as geometric means with corresponding standard deviations. Significant differences are indicated. LFCs = limbal fibroblast cells (n = 7), AN-LFCs = aniridia limbal fibroblast cells (n = 7).

However, a noticeable rise in PAX6 mRNA expression occurred after 24 h exposure to LPS, resulting in an approximate fivefold increase (FC_17.5 µg/ml LPS_ = 5.119) for LFCs (*p* = 0.020) and elevenfold increase (FC_17.5 µg/ml LPS_ = 6.129) for AN-LFCs (*p* < 0.0001), relative to their untreated baseline levels (Fig 1A). In contrast, OS exerted no discernible impact on PAX6 expression after 48 h treatment (Fig 1B).

Unlike in corneal epithelial and conjunctival cells, the presence of PAX6 protein could not be confirmed under any of the examined conditions via western blot analysis (S1 Fig).

### Effect of LPS-inflammation on cytokine expression

**Fig 2:**
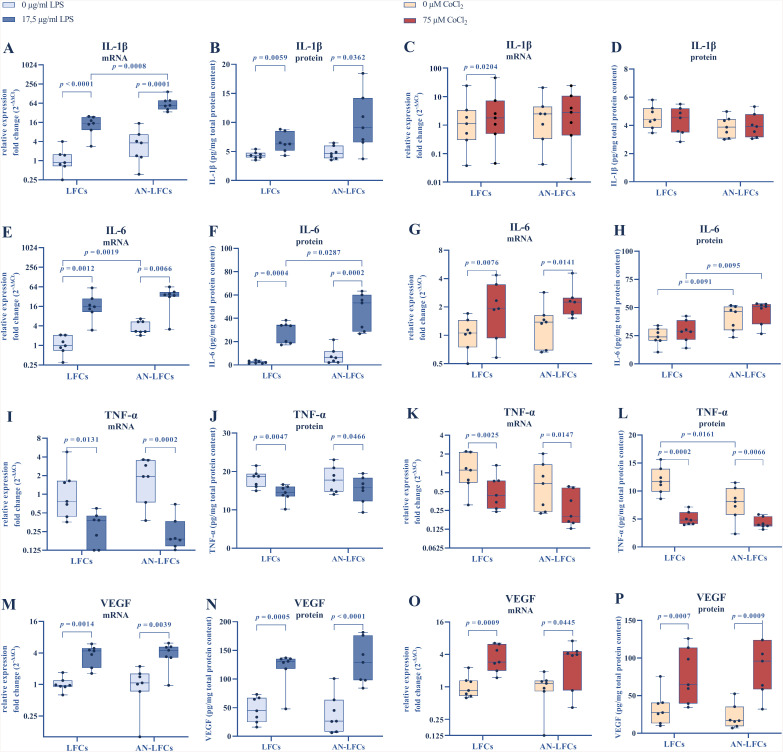
Effect of LPS-induced inflammation (blue) and CoCl_2_-triggered oxidative stress (red) on cytokine expression. IL-1α **(A–D)**, IL-6 **(E–H)**, TNF-α **(I–L)**, and VEGF (M–P) mRNA and protein expression. The induction of an inflammatory response was achieved by applying 17.5 µg/ml E. coli LPS over a 24 h period. Oxidative stress was simulated by administering 75 µM CoCl_2_ over 48 **h.** Results are presented as geometric means for mRNA expression and arithmetic means for protein levels, accompanied by corresponding standard deviations. Significant differences are indicated. LFCs = limbal fibroblast cells (n = 7), AN-LFCs = aniridia limbal fibroblast cells (n = 7).

LPS treatment induced IL-1β upregulation at both mRNA and protein levels in LFCs (*p* < 0.0001, *p* = 0.006) as well as AN-LFCs (*p* = 0.0001, *p* = 0.036). Under LPS conditions, AN-LFCs exhibited a significant higher IL-1β mRNA expression compared to LFCs (*p* = 0.0008). This trend was also noticeable in protein measurements, although statistical significance was not achieved (*p* = 0.124) (Fig 2A, B).

Untreated AN-LFCs already exhibited a higher IL-6 mRNA level than normal LFCs (*p* = 0.002). Moreover, AN-LFCs secreted approximately three times more IL-6 in protein, however, without statistically significant impact (*p* = 0.091). Under LPS exposure, IL-6 increased in gene and protein expression in both LFCs (*p* = 0.001, *p* = 0.0004) and AN-LFCs (*p* = 0.007, *p* = 0.0002), with AN-LFCs producing more IL-6 protein than normal LFCs under LPS conditions (*p* = 0.029) (Fig 2E, F).

In the course of LPS-induced inflammation, TNF-α exhibited a significant decrease at both gene and protein levels in LFCs (*p* = 0.013, *p* = 0.005) and AN-LFCs (*p* = 0.0002, *p* = 0.047). However, no differences were observed between LFCs and AN-LFCs (Fig 2I, J).

The VEGF gene and protein expression increased in LFCs (*p* = 0.001, *p* = 0.0005) and AN-LFCs (*p* = 0.004, *p* < 0.0001) following LPS treatment. No difference under any conditions were found between LFCs and AN-LFCs (Fig 2M, N).

### Effect of CoCl_2_ induced oxidative stress on cytokine expression

CoCl_2_ treatment, unlike LPS, did not induce expressional changes in IL-1β, except for mRNA levels in LFCs (*p* = 0.020) (Fig 2C, D).

On the other hand, IL-6 mRNA increased significantly with OS in LFCs (*p* = 0.008) and AN-LFCs (*p* = 0.014), whereas the corresponding protein levels showed only a non-significant trend toward an increase in LFCs (*p* = 0.057) and AN-LFCs (*p* = 0.074). At protein level, AN-LFCs exhibited higher IL-6 expression, both untreated (*p* = 0.009) and after CoCl_2_ exposure (*p* = 0.010) (Fig 2G, H).

TNF-α levels decreased on both mRNA and protein levels in response to CoCl_2_ treatment in LFCs (*p* = 0.003, *p* = 0.0002) and AN-LFCs (*p* = 0.015, *p* = 0.007). Moreover, untreated AN-LFCs demonstrated a reduced TNF-α expression at protein level (*p* = 0.016), compared to normal LFCs (Fig 2K, L).

VEGF expression significantly increased in terms of both gene and protein under OS in LFCs (*p* = 0.0009, *p* = 0.0007) and AN-LFCs (*p* = 0.045, *p* = 0.0009). There was no discernible difference between the two groups across any of the studied conditions (Fig 2O, P).

## Discussion

One of the important questions in AAK research remains the degree to which the disease arises from deficiencies of LESC themselves, or whether the pathomechanism is predominantly driven by alterations of the stem cell niche [15]. Already at a very early stage of the disease, the limbal stroma exhibits fundamental perturbances, along with a massive immune cell infiltration that appears to precede the structural breakdown of Vogt’s palisades [7,15,19]. Therefore, some authors suspect that the observed morphological changes in the stem cell niche could result from proteolytic degradation in the context of ongoing inflammation [15]. As cytokines can also directly influence the differentiation and migration processes of LESCs [4,34], many of the abnormalities observed in AAK may potentially be attributed to the chronic inflammatory state [35].

In this study, we explored PAX6 expression, along with the cytokines IL-1β, IL-6, TNF-α, and VEGF in LFCs from corneal donors, compared to specimens obtained from aniridia patients under *in vitro* conditions.

Our findings revealed alterations in gene and protein expression linked to inflammation in untreated AN-LFCs, with a marked increase in IL-6 mRNA and protein levels compared to normal LFCs. Furthermore, we subjected LFCs and AN-LFCs to LPS and CoCl_2_ treatment to induce a specific inflammatory reaction. Following treatment, AN-LFCs demonstrated elevated IL-1β mRNA and IL-6 protein levels, compared to normal LFCs. These results suggest a potential involvement of LFCs in the inflammatory processes observed in AAK.

### PAX6 transcription factor

PAX6 is a highly conserved transcription factor, presumed to be involved in the regulation of over 2000 downstream genes and numerous processes of ocular morphogenesis and corneal homeostasis [19,36]. As most cases of congenital aniridia result from heterozygote mutation in PAX6 gene [5], this can explain the diverse manifestations of the condition. The haploinsufficiency observed in corneal epithelial cells of aniridia patients leads to an attainment of only 60–70% of the physiological PAX6 expression [3]. Similarly, our untreated AN-LFCs exhibited, on average, an equivalent reduction in gene expression compared to normal LFCs (57–72%). The low absolute PAX6 protein expression in corneal fibroblasts, as demonstrated by Li et al. [37], posed an additional difficulty, making it impossible to capture protein levels through Western blot analysis, irrespective of the experimental condition.

It is well known that PAX6 is involved in corneal epithelial wound healing and undergoes upregulation in the regeneration process after mechanical or thermal injury [18,38]. Interestingly, our LFCs also exhibited a significant upregulation of PAX6 gene expression after LPS treatment, suggesting that the constitutively low transcript levels in fibroblasts can be increased to a possibly relevant extent when needed. A noteworthy observation was that the OS induction using CoCl_2_ resulted, as expected, in an inflammatory response; however, unlike the treatment with LPS, it did not lead to an enhancement of PAX6 expression. A contributing factor to this could be the regulatory influence of OS on PAX6, working against its upregulation during the inflammatory state. Notably, OS-induced changes in posttranslational modification of a DNA-binding factor is known to block the PAX6 promoter region [39]. Moreover, OS induces nuclear-cytoplasmic translocation of PAX6 [23]. Given that the transcription factor possesses an autoregulatory DNA-binding site [19], enhancing its own expression, this mechanism could counteract the increased PAX6 expression during inflammation. Considering that inflammation is always associated with tissue destruction, requiring subsequent wound healing, this mechanism appears dysfunctional. It is known that PAX6 haploinsufficient cells exhibit increased sensitivity to OS [23]. Through the described mechanisms, the PAX6 deficit in AN-LFCs under OS could be further exacerbated, which was observed here on average, although not statistically significant (FC_AN-LFC, 75µM CoCl2_ = 0.53).

### Cytokine expression in untreated LFCs and AN-LFCs

In the tear film of aniridia patients, a markedly elevated cytokine concentration has been consistently observed [40]. To our knowledge, we are the first to document a similar observation in cell culture. This is remarkable as it demonstrates that limbal fibroblasts from aniridia patients have an intrinsic tendency for increased cytokine secretion, even after multiple cell passages and in an “optimized” environment that shields them from other influences that could *in vivo* be responsible for the inflammatory state in AAK. In comparison to normal LFCs, the untreated AN-LFCs expressed significantly higher levels of IL-6, both at the mRNA and protein levels. IL-6 is one of the key molecules in the inflammatory process and plays a crucial role in various ocular pathologies such as glaucoma, keratoconjunctivitis sicca, as well as corneal neovascularization and fibrosis [41]. Hence it could contribute to the fibrotic remodeling within the stroma seen in AAK. Less recognized, however, is that IL-6 also serves as a central messenger in epithelial-stromal communication, influencing the epithelial expression of stem cell and differentiation markers. As such, IL-6 plays a pivotal role in maintaining the progenitor characteristics of limbal epithelial cells [34]. Considering that epithelial cells from aniridia patients lack crucial structural proteins, which are typically upregulated during differentiation [20], there is an alignment with the expected impact of an IL-6 excess, leading to an immature morphology and reduced keratin-3 expression [34]. Furthermore, IL-6 is known to boost proliferation in epidermal keratinocytes across several cell generations [42]. It also speeds up the *in vitro* migration of corneal epithelial cells [43] and epithelial wound closure [44], hinting at its potential role in the accelerated healing response, that is observed in aniridia whole organ cell models [14,18].

### Cytokine expression in LFCs and AN-LFCs during inflammation and oxidative stress

Under LPS treatment, IL-1β, IL-6, and VEGF at both mRNA and protein levels increased. Similar effects were observed with OS induced by CoCl_2_. Notably, the increase in IL-1β with OS was only apparent at the mRNA level in LFCs. Since IL-1β, along with TNF-α, is typically upregulated early in the inflammatory process, this can potentially be explained by the time discrepancy between the 24 h LPS and 48 h CoCl_2_ exposure. Both LPS and CoCl_2_ caused a decrease in TNF-α, which we considered as compensatory regulation, following an earlier increase. This increase was observed with a shorter treatment duration, particularly at 3 hours (unpublished data). Tolerance to LPS-stimulation has been reported for TNF-α in a monocyte cell line. However, this did not affect IL-1β, while IL-6 even intensified with repeated LPS exposure [45], emphasizing that cytokine secretion undergoes a highly complex time-dependent regulation, complicating the choice of an appropriate measurement point.

With LPS exposure, AN-LFCs exhibited higher IL-1β mRNA as well as IL-6 protein expression compared to normal LFCs, which was validated by an elevated IL-6 protein secretion under OS.

## Limitations

One limitation of our study is the difficulty of extrapolating findings derived from cell culture conditions to *in vivo* situations. This challenge is particularly evident because the limbal stem cell niche, from which we isolated the LFCs, presents a highly intricate microenvironment, contrasting with the central cornea [19]. In addition to the interaction with the vascular and nervous system, this environment naturally involves a close crosstalk with local melanocytes and immune cells [19,46]. These interactions collectively constitute influences that have been dismissed in our cell culture experiments. Moreover, the influence of FCS during cell propagation led to a differentiation of isolated keratocytes into fibroblasts, a factor that likely had a considerable impact on gene and protein expression. Due to the limited availability of primary cells from aniridia patients, we could only investigate a relatively small sample size, making it challenging to demonstrate existing differences statistically.

## Conclusions

In summary, the present study provides evidence that AN-LFCs secrete higher IL-6 levels than cells from unaffected corneal donors, even in the absence of specific inflammatory stimuli. This finding extends to situations where AN-LFCs are exposed to inflammatory triggers such as LPS, a component of bacterial cell membranes, or CoCl_2_ as a chemical OS inductor. Furthermore, an increased IL-1β mRNA expression in AN-LFCs compared to normal LFCs was detected under LPS exposure. This suggests a potential involvement of LFCs in the inflammatory processes observed in AAK. Moreover, we explored the expression of the transcription factor PAX6 in LFCs and AN-LFCs, which was too minimal to be detected at protein level via Western blot analysis. On average, AN-LFCs exhibited lower PAX6 mRNA expression, though not reaching statistical significance. Notably, under LPS treatment, there was a significant increase in PAX6 expression, which was not seen with CoCl_2_ treatment. Since inflammatory processes are a crucial component of AAK, and fibroblasts are considered as sentinel cells that induce and modulate these processes, further experiments are needed to understand the role of limbal fibroblasts in the development of AAK.

## Supporting information

S1 Table Protein expression levels of the interleukins IL-1β and IL-6, tumor necrosis factor-α (TNF-α), and vascular endothelial growth factor (VEGF) in limbal fibroblast cells of corneal donors (LFCs) and aniridia patients (AN-LFCs), both untreated and following induction of inflammation (via LPS) and oxidative stress (via CoCl₂). All treatments were performed on cells derived from the same biological replicate (i.e., the same donor and passage). Different LPS- and CoCl_2_-concentrations were applied in parallel as treatment conditions within each replicate. A total of seven (*n* = 7) independent biological replicates were included per group (LFCs and AN-LFCs). The concentrations of the proteins of interest in the cell culture supernatants were normalized to the total protein content of the corresponding cell lysates, yielding values expressed in picograms per milligram of total protein. Data are presented as mean ± standard deviation. The raw data for all individual measurements corresponding to the mean values are reported in S1 Dataset.
(DOCX)

S2 Table mRNA expression levels of PAX6, the interleukins IL-1β and IL-6, tumor necrosis factor-α (TNF-α), and vascular endothelial growth factor (VEGF) in limbal fibroblast cells from corneal donors (LFCs) and aniridia patients (AN-LFCs), both untreated and following induction of inflammation (via LPS) and oxidative stress (via CoCl₂). All treatments were performed on cells derived from the same biological replicate (i.e., the same donor and passage). Different LPS- and CoCl_2_-concentrations were applied in parallel as treatment conditions within each replicate. A total of seven (*n* = 7) independent biological replicates were included per group (LFCs and AN-LFCs). Expression levels are presented as fold changes relative to the untreated LFCs and are reported as geometric mean ± geometric standard deviation. The raw data for all individual measurements corresponding to the mean values are reported in S2 Dataset.
(DOCX)

S1 Fig Representative Western blot for detection of PAX6 protein in untreated and treated limbal fibroblasts, compared to conjunctival and epithelial cells. This figure shows Western blot membranes from experiments using LPS (A) and cobalt chloride (B) treatments in limbal fibroblasts derived from corneal donors (LFCs) and aniridia patients (AN-LFCs). No detectable PAX6 protein expression (expected molecular weight: 46 and 48 kDa) was observed in these samples. Protein lysates from conjunctival tissue and epithelial cells were used as positive controls, showing clear PAX6 expression in contrast to the limbal fibroblasts. The procedure was performed as described in Trusen et al. [32]. Briefly, the cell pellets were lysed in RIPA buffer (Sigma-Aldrich, St. Louis, USA), and total protein concentrations were measured using the Pierce^™^ BCA Protein Assay Kit (Thermo Fisher Scientific, Waltham, USA) with a Tecan Infinite F50 Absorbance Microplate Reader (Tecan Group AG, Männedorf, Switzerland) at 560 nm wavelength. For Western blotting, 20 μg of total protein per sample was denatured in 4 × Laemmli Sample Buffer (Bio-Rad Laboratories, Hercules, CA, USA) at 95°C for 5 minutes. Proteins were separated on Invitrogen^™^ NuPAGE^™^ 4–12% Bis-Tris Mini Gels (Thermo Fisher Scientific) using NuPAGE^™^ MOPS SDS Running Buffer (Thermo Fisher Scientific), and transferred onto nitrocellulose membranes using the Trans-Blot^®^ Turbo^™^ Transfer System (Bio-Rad Laboratories). Total protein normalization (TPN) was performed using Invitrogen^™^ No-Stain^™^ Protein Labeling Reagent (Thermo Fisher Scientific). Membranes were washed with hypotonic water and WesternFroxx Wash Solution (neoFroxx GmbH, Einhausen, Germany), then incubated with primary antibodies (Cell Signaling Technology) diluted in WesternFroxx Solution B (neoFroxx), which also contained blocking reagent and HRP-conjugated secondary antibody. After further washing, membranes were exposed to the Western Lightning^®^ Plus ECL Reagent (PerkinElmer, Inc., Waltham, USA) for for detection by chemiluminescence. Both the chemiluminescence signal and the TPN labeling were imaged using the Invitrogen^™^ iBright^™^ CL1500 Imaging System (Thermo Fisher Scientific), and densitometric analysis was performed using the provided software.
(DOCX)

S1 Dataset Raw data ELISA. This file lists the raw data for all individual measurements corresponding to the mean values reported in S1 Table. (XLSX)

S2 Dataset Raw data qPCR. This file lists the raw data for all individual measurements corresponding to the mean values reported in S2 Table. (XLSX)
