# Supplementary material for: Proinflammatory gene and protein expression alterations in human limbal aniridia fibroblasts
Source: PLoS One. 2025 Dec 4;20(12):e0337114. doi: 10.1371/journal.pone.0337114 (PMC12677563; doi:10.1371/journal.pone.0337114)
Supplement: S1 Table — All treatments were performed on cells derived from the same biological replicate (i.e., the same donor and passage). Different LPS- and CoCl2-concentrations were applied in parallel as treatment conditions within each replicate. A total of seven (n = 7) independent biological replicates were included per group (LFCs and AN-LFCs). The concentrations of the proteins of interest in the cell culture supernatants were normalized to the total protein content of the corresponding cell lysates, yielding values expressed in picograms per milligram of total protein. Data are presented as mean ± standard deviation. The raw data for all individual measurements corresponding to the mean values are reported in S1 Dataset. (DOCX) [file pone.0337114.s001.docx]

| **protein** | **Limbal fibroblast cells of corneal donors (LFCs) and aniridia patients (AN-LFCs): protein expression (pg target / mg of total protein)** | | | | | | | |
| --- | --- | --- | --- | --- | --- | --- | --- | --- |
|  | **LPS-induced inflammation** | | | | **CoCl_2_-triggered oxidative stress** | | | |
|  | **LFC** | | **AN-LFC** | | **LFC** | | **AN-LFC** | |
|  | **0 µg/ml**  **LPS** | **17,5 µg/ml**  **LPS** | **0 µg/ml**  **LPS** | **17,5 µg/ml LPS** | **0 µM**  **CoCl_2_** | **75 µM**  **CoCl_2_** | **0 µM**  **CoCl_2_** | **75 µM**  **CoCl_2_** |
| IL-1β | 4.36 ± 0.61 | 6.55 ± 1.65 | 4.79 ± 1.10 | 10.02 ± 5.01 | 4.56 ± 0.82 | 4.29 ± 1.00 | 3.89 ± 0.74 | 3.98 ± 0.84 |
| IL-6 | 2.44 ± 1.12 | 28.11 ± 8.95 | 7.77 ± 6.98 | 45.78 ± 15.78 | 24.20 ± 7.83 | 29.19 ± 9.66 | 40.66 ± 11.25 | 45.88 ± 10.57 |
| TNF-α | 18.04 ± 2.25 | 14.33 ± 2.15 | 17.82 ± 3.39 | 15.29 ± 3.52 | 11.90 ± 2.39 | 5.05 ± 1.20 | 7.75 ± 3.07 | 4.28 ± 0.99 |
| VEGF | 46.31 ± 22.47 | 119.02 ± 31.96 | 39.52. ± 33.74 | 130.10 ± 38.84 | 33.14 ± 22.06 | 76.63 ± 36.26 | 21.97 ± 16.21 | 91.23 ± 43.26 |

**S1 Table. Protein expression levels of the interleukins IL-1β and IL-6, tumor necrosis factor-α (TNF-α), and vascular endothelial growth factor (VEGF) in limbal fibroblast cells of corneal donors (LFCs) and aniridia patients (AN-LFCs), both untreated and following induction of inflammation (via LPS) and oxidative stress (via CoCl₂).** All treatments were performed on cells derived from the same biological replicate (i.e., the same donor and passage). Different LPS- and CoCl_2_-concentrations were applied in parallel as treatment conditions within each replicate. A total of seven (*n* = 7) independent biological replicates were included per group (LFCs and AN-LFCs). The concentrations of the proteins of interest in the cell culture supernatants were normalized to the total protein content of the corresponding cell lysates, yielding values expressed in picograms per milligram of total protein. Data are presented as mean ± standard deviation. The raw data for all individual measurements corresponding to the mean values are reported in S1 Dataset.
