# Supplementary material for: Proinflammatory gene and protein expression alterations in human limbal aniridia fibroblasts
Source: PLoS One. 2025 Dec 4;20(12):e0337114. doi: 10.1371/journal.pone.0337114 (PMC12677563; doi:10.1371/journal.pone.0337114)
Supplement: S2 Table — All treatments were performed on cells derived from the same biological replicate (i.e., the same donor and passage). Different LPS- and CoCl2-concentrations were applied in parallel as treatment conditions within each replicate. A total of seven (n = 7) independent biological replicates were included per group (LFCs and AN-LFCs). Expression levels are presented as fold changes relative to the untreated LFCs and are reported as geometric mean ± geometric standard deviation. The raw data for all individual measurements corresponding to the mean values are reported in S2 Dataset. (DOCX) [file pone.0337114.s002.docx]

| **gene** | **Limbal fibroblast cells of corneal donors (LFCs) and aniridia patients (AN-LFCs):  geometric mean and standard deviation of fold changes (2^-ΔΔCT^)** | | | | | | | |
| --- | --- | --- | --- | --- | --- | --- | --- | --- |
|  | **LPS-induced inflammation** | | | | **CoCl_2_-triggered oxidative stress** | | | |
|  | **LFCs** | | **AN-LFCs** | | **LFCs** | | **AN-LFCs** | |
|  | **0 µg/ml**  **LPS** | **17,5 µg/ml**  **LPS** | **0 µg/ml**  **LPS** | **17,5 µg/ml LPS** | **0 µM**  **CoCl_2_** | **75 µM**  **CoCl_2_** | **0 µM**  **CoCl_2_** | **75 µM**  **CoCl_2_** |
| PAX6 | 1.00 ± 3.34 | 5.12 ± 3.87 | 0.57 ± 3.01 | 6.13 ± 2.95 | 1.00 ± 3.04 | 1.07 ± 2.82 | 0.71 ± 1.92 | 0,53 ± 3.94 |
| IL-1β | 1.00 ± 2.40 | 12.82 ± 2.10 | 2.69 ± 3.36 | 61.71 ± 1.62 | 1.00 ± 7.60 | 1.67 ± 8.67 | 1.35 ± 7.30 | 1.54 ± 11.97 |
| IL-6 | 1.00 ± 1.97 | 15.26 ± 2.50 | 3.57 ± 1.59 | 28.01 ± 2.69 | 1.00 ± 1.57 | 1.83 ± 2.03 | 1.29 ± 1.66 | 2.22 ± 1.44 |
| TNF-α | 1.00 ± 2.46 | 0.26 ± 2.14 | 1.69 ± 2.35 | 0.23 ± 1.81 | 1.00 ± 1.99 | 0.49 ± 1.87 | 0.60 ± 2.40 | 0.26 ± 1.90 |
| VEGF | 1.00 ± 1.35 | 3.56 ± 1.62 | 0.72 ± 4.57 | 3.61 ± 1.87 | 1.00 ± 1.59 | 3.32 ± 1.77 | 0.68 ± 4.51 | 2.57 ± 2.84 |

**S2 Table. mRNA expression levels of PAX6, the interleukins IL-1β and IL-6, tumor necrosis factor-α (TNF-α), and vascular endothelial growth factor (VEGF) in limbal fibroblast cells from corneal donors (LFCs) and aniridia patients (AN-LFCs), both untreated and following induction of inflammation (via LPS) and oxidative stress (via CoCl₂).** All treatments were performed on cells derived from the same biological replicate (i.e., the same donor and passage). Different LPS- and CoCl_2_-concentrations were applied in parallel as treatment conditions within each replicate. A total of seven (*n* = 7) independent biological replicates were included per group (LFCs and AN-LFCs). Expression levels are presented as fold changes relative to the untreated LFCs and are reported as geometric mean ± geometric standard deviation. The raw data for all individual measurements corresponding to the mean values are reported in S2 Dataset.
