# Supplementary material for: Proinflammatory gene and protein expression alterations in human limbal aniridia fibroblasts
Source: PLoS One. 2025 Dec 4;20(12):e0337114. doi: 10.1371/journal.pone.0337114 (PMC12677563; doi:10.1371/journal.pone.0337114)
Supplement: S1 Fig — This figure shows Western blot membranes from experiments using LPS (A) and cobalt chloride (B) treatments in limbal fibroblasts derived from corneal donors (LFCs) and aniridia patients (AN-LFCs). No detectable PAX6 protein expression (expected molecular weight: 46 and 48 kDa) was observed in these samples. Protein lysates from conjunctival tissue and epithelial cells were used as positive controls, showing clear PAX6 expression in contrast to the limbal fibroblasts. The procedure was performed as described in Trusen et al. [32]. Briefly, the cell pellets were lysed in RIPA buffer (Sigma-Aldrich, St. Louis, USA), and total protein concentrations were measured using the Pierce™ BCA Protein Assay Kit (Thermo Fisher Scientific, Waltham, USA) with a Tecan Infinite F50 Absorbance Microplate Reader (Tecan Group AG, Männedorf, Switzerland) at 560 nm wavelength. For Western blotting, 20 μg of total protein per sample was denatured in 4 × Laemmli Sample Buffer (Bio-Rad Laboratories, Hercules, CA, USA) at 95°C for 5 minutes. Proteins were separated on Invitrogen™ NuPAGE™ 4–12% Bis-Tris Mini Gels (Thermo Fisher Scientific) using NuPAGE™ MOPS SDS Running Buffer (Thermo Fisher Scientific), and transferred onto nitrocellulose membranes using the Trans-Blot® Turbo™ Transfer System (Bio-Rad Laboratories). Total protein normalization (TPN) was performed using Invitrogen™ No-Stain™ Protein Labeling Reagent (Thermo Fisher Scientific). Membranes were washed with hypotonic water and WesternFroxx Wash Solution (neoFroxx GmbH, Einhausen, Germany), then incubated with primary antibodies (Cell Signaling Technology) diluted in WesternFroxx Solution B (neoFroxx), which also contained blocking reagent and HRP-conjugated secondary antibody. After further washing, membranes were exposed to the Western Lightning® Plus ECL Reagent (PerkinElmer, Inc., Waltham, USA) for for detection by chemiluminescence. Both the chemiluminescence signal and the TPN labeling were imaged using the Invitrogen™ iBrigh [file pone.0337114.s003.docx]

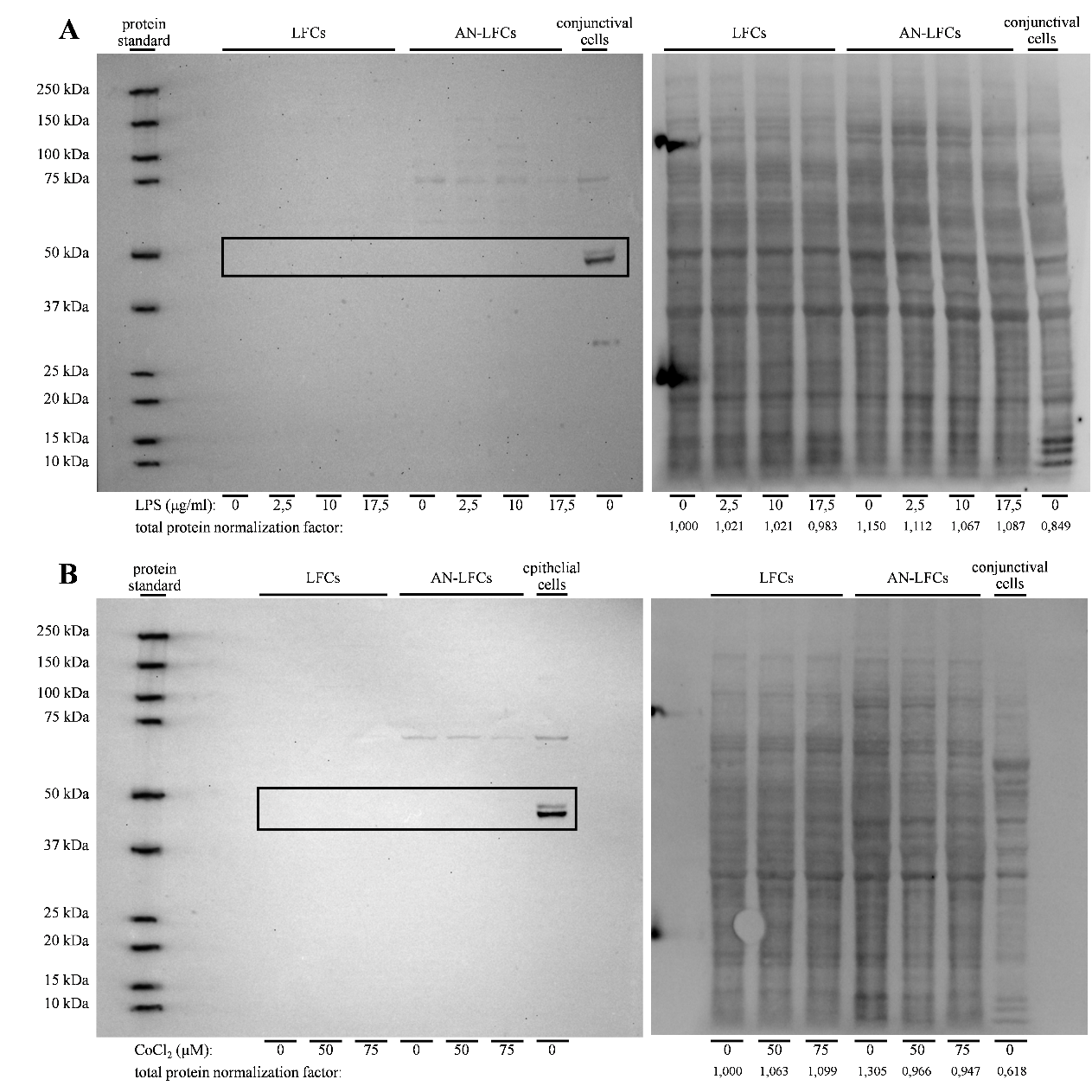


**S1 Fig. Representative Western blot for detection of PAX6 protein in untreated and treated limbal fibroblasts, compared to conjunctival and epithelial cells.** This figure shows Western blot membranes from experiments using LPS (A) and cobalt chloride (B) treatments in limbal fibroblasts derived from corneal donors (LFCs) and aniridia patients (AN-LFCs). No detectable PAX6 protein expression (expected molecular weight: 46 and 48 kDa) was observed in these samples. Protein lysates from conjunctival tissue and epithelial cells were used as positive controls, showing clear PAX6 expression in contrast to the limbal fibroblasts. The procedure was performed as described in Trusen et al. [32].
Briefly, the cell pellets were lysed in RIPA buffer (Sigma-Aldrich, St. Louis, USA), and total protein concentrations were measured using the Pierce^™^ BCA Protein Assay Kit (Thermo Fisher Scientific, Waltham, USA) with a Tecan Infinite F50 Absorbance Microplate Reader (Tecan Group AG, Männedorf, Switzerland) at 560 nm wavelength. For Western blotting, 20 μg of total protein per sample was denatured in 4× Laemmli Sample Buffer (Bio-Rad Laboratories, Hercules, CA, USA) at 95 °C for 5 minutes. Proteins were separated on Invitrogen^™^ NuPAGE^™^ 4–12% Bis-Tris Mini Gels (Thermo Fisher Scientific) using NuPAGE^™^ MOPS SDS Running Buffer (Thermo Fisher Scientific), and transferred onto nitrocellulose membranes using the Trans-Blot^®^ Turbo^™^ Transfer System (Bio-Rad Laboratories). Total protein normalization (TPN) was performed using Invitrogen^™^ No-Stain^™^ Protein Labeling Reagent (Thermo Fisher Scientific). Membranes were washed with hypotonic water and WesternFroxx Wash Solution (neoFroxx GmbH, Einhausen, Germany), then incubated with primary antibodies (Cell Signaling Technology) diluted in WesternFroxx Solution B (neoFroxx), which also contained blocking reagent and HRP-conjugated secondary antibody. After further washing, membranes were exposed to the Western Lightning^®^ Plus ECL Reagent (PerkinElmer, Inc., Waltham, USA) for for detection by chemiluminescence. Both the chemiluminescence signal and the TPN labeling were imaged using the Invitrogen^™^ iBright^™^ CL1500 Imaging System (Thermo Fisher Scientific), and densitometric analysis was performed using the provided software.
